# Brain inspired path planning algorithms for drones

**DOI:** 10.3389/fnbot.2023.1111861

**Published:** 2023-03-03

**Authors:** Yixun Chao, Philipp Augenstein, Arne Roennau, Ruediger Dillmann, Zhi Xiong

**Affiliations:** ^1^Navigation Research Center, School of Automation Engineering in Nanjing University of Aeronautics and Astronautics, Nanjing, China; ^2^FZI Research Center for Information Technology, Karlsruhe, Germany

**Keywords:** path planning, navigation, place cells, spiking neural network, Airsim

## Abstract

**Introduction:**

With the development of artificial intelligence and brain science, brain-inspired navigation and path planning has attracted widespread attention.

**Methods:**

In this paper, we present a place cell based path planning algorithm that utilizes spiking neural network (SNN) to create efficient routes for drones. First, place cells are characterized by the leaky integrate-and-fire (LIF) neuron model. Then, the connection weights between neurons are trained by spike-timing-dependent plasticity (STDP) learning rules. Afterwards, a synaptic vector field is created to avoid obstacles and to find the shortest path.

**Results:**

Finally, simulation experiments both in a Python simulation environment and in an Unreal Engine environment are conducted to evaluate the validity of the algorithms.

**Discussion:**

Experiment results demonstrate the validity, its robustness and the computational speed of the proposed model.

## 1. Introduction

A drone, also called an unmanned aerial vehicle (UAV), is a vehicle capable to perform autonomous motion decisions or human like remote control. Due to the high mobility, flexibility, and low cost, drones have a wide range of military and civilian applications such as target detection, epidemic prevention, disaster relief and so on (Mohsan et al., 2022). Autonomous flight of a drone includes navigation, simultaneous localization and mapping as well as path planning. Path planning involves calculating an efficient route from a launch position to a target while avoiding obstacles, which plays an important role in the task execution of a drone (Aggarwal and Kumar, 2020). In practical applications, many flight tasks of UAV are in static environment, such as cargo transportation in a given environment, military attack driven by interest targets and so on. In these circumstances, it is particularly important to develop path planning algorithms for drones in static environments, and it also puts forward higher requirements for the intelligence, reliability and autonomy of path planning methods. Despite great progress in decades of drone research, there are still many open questions for path planners.

The conventional global path planning algorithms mainly include artificial potential field methods, A^*^ algorithm, ant colony algorithms, rapidly exploring random trees (RRT), and optimization and improvement algorithms of these algorithms (Noreen et al., 2016; Konatowski and Pawłowski, 2018; Cai et al., 2019; Mohammed et al., 2021). However, all these path planning algorithms need to model the environment in advance, which is not suitable for the problem of global path pre-planning of drones in complex environment. As neuroscience gradually reveals how the brain represents spatial information, the effort to construct biologically-inspired drone controllers that utilize these spatial representations has become feasible. Of particular interests in this context are place cells, which are assumed to be responsible for spatial representation (Leutgeb et al., 2005; Yartsev and Ulanovsky, 2013). Inspired by these advances, many neural models based on place cells have been proposed to enable an agent to find its way to a destination. Zannone et al. presented a reward-driven goal-directed method modeling place cells and action cells (Zannone et al., 2018). Koul et al. introduced a spiking neural network model and a neuromorphic implementation for path planning inspired by place cells that use spike latency in the pathfinding process (Koul and Horiuchi, 2019). Zennir proposed a robust path planning algorithm by propagating rhythmic spiking activity in a hippocampal network model (Zennir et al., 2017). Although all the above methods can guide an agent to a target and represent path planning algorithms in two-dimension (2D) environments. There is increasing interest on biologically-inspired path planning algorithms for drones in three- dimensional (3D) space. However, there are only a few reports addressing neural brain like approaches for 3D path planning.

To bridge the major gap of existing studies, we propose a biologically plausible 3D model of a spiking neural network (SNN) that enables fast and reliable computation of a feasible pathway of a drone. The neural representation of place cells receives information about the initial position of the drone and performs position updates through neural plasticity. Obstacles are represented by inhibited neurons. When the network receives the coordinates of a target location, it is able to steer the drone toward a target based on a synaptic vector field. Finally, the feasibility of the model is to be verified by simulation experiments and Airsim. The rest of the paper is organized as follows: Section 2 describes the overall proposed framework, Section 3 depicts the implemented system and the proposed path planning algorithm, while Section 4 presents the experimental setup and simulation results, which are then summarized in Section 5.

## 2. Network architecture

**Figure 1:**
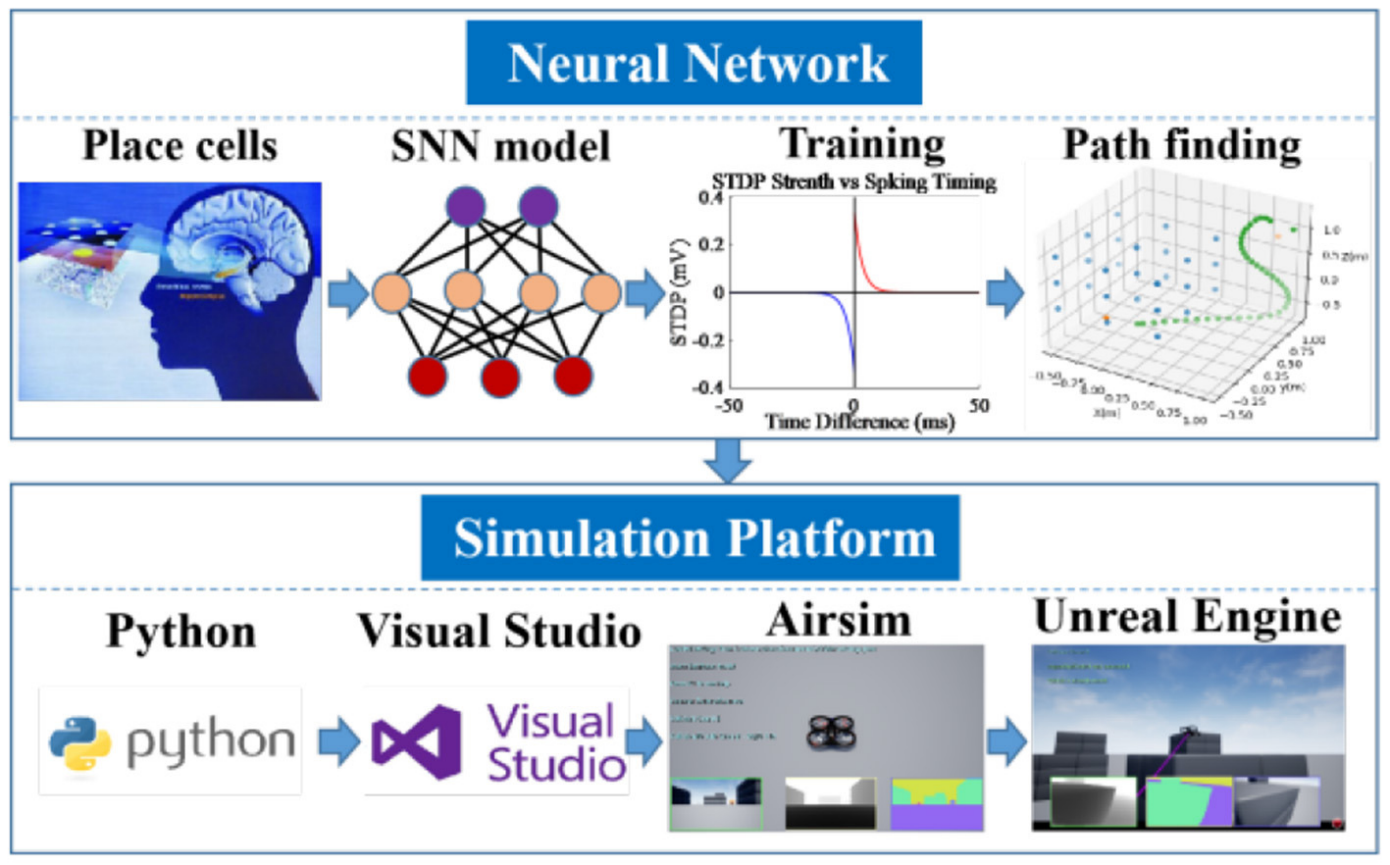
The architecture of brain-inspired path planning.

The goal of this work is to design and implement a complete navigation system for drones in which most of the computation takes place in neural networks that use spatial representations similar to place cells in the brain of mammals. The drone should be able to compute feasible trajectories to targets in a complex environment with obstacles. In addition, the drone should be able to learn all necessary information about its environment by exploration. Finally, the algorithm is verified with the help of Airsim and the Unreal Engine. The overall architecture of the path planning system is shown in Figure 1.

**Figure 2:**
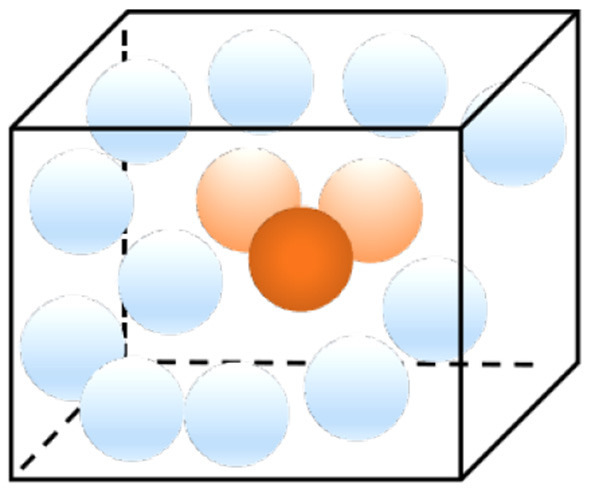
The firing rate map of 3D place cells.

Place cells are a type of pyramidal neurons in the hippocampus that become active once an animal enters a specific location in its environment, called a place field. Place cells are assumed to form the basis of an internal representation of a particular position in space, known as a cognitive map (Burgess and O'Keefe, 1996). It is reported that the place cells in the hippocampus of the brain generate sequences that predict the future path when the animal explores the environment, which suggests that the hippocampus operates similarly to a GPS unit that shows not only where one currently is, but also how to reach the destination (Schmidt and Redish, 2013). 3D place cells in the mammalian hippocampal formation imply and support the existence of 3D cognitive maps. Figure 2 shows the firing representation of place cells to different regions (Soman et al., 2018). Place cells are filled throughout the spatial environment. The closer the cells are to the current position of the drone, the higher the firing rate of the place cells. In Figure 2, the red cell has the highest firing rate, while the lighter part has a lower firing rate. The cooperative firing of all place cells forms the basis of a spatial cognitive map.

One promising approach to duplicate brain-inspired behavior is to mimic the underlying neural activity. Recent developments of large-scale neuromorphic hardware offering unprecedented asynchronous parallelism and energy efficiency such as the Intel Loihi, the IBM True North, and the SpiNNaker, have further advanced the development of SNN-based robotic controls (Xin et al., 2010; Lin et al., 2018; Löhr et al., 2020). However, despite the emerging interest in autonomous robot systems, research on SNN-controlled 3D drone navigation is still in its early exploratory stage. SNN-driven autonomous control systems for drones, capable of handling complex scenarios in a robust and natural way, are particularly needed. Spike-timing-dependent plasticity (STDP) occurs in synapses of hippocampal place cells. A classic STDP rule fits well with the synaptic information transfer mode. In this study, a SNN-based place cell model is applied to develop a biologically constrained brain- inspired navigation system. The connection weights between cells are trained with the help of STDP (Hao et al., 2020). On this basis we propose a neuromorphic navigation method for autonomous drones being capable to generate biologically plausible goal-directed behaviors.

A virtual drone platform is used to perform in simulation mode the required flight tests without damaging or harming a real drone, which allows improving training efficiency and saving training costs (Wang et al., 2017). Airsim is a widely popular virtual simulator due to its ability to interact with drones in simulation programs through languages such as Python, cross-platform flight control through flight controllers, and physically and visually realistic simulation environments.

## 3. Navigation with neural networks

To enable the drone to navigate to destinations that are not necessarily accessible by the shortest route from the current location, route planning is required. Here, a biologically plausible path planner is proposed for drones based on SNNs, where each neuron is modeled according the leaky integrate-and-fire model (LIF) and represents a place cell, as a basic unit of the discrete flying environment (Escamilla, 2006). The main processing steps are as follows: (1) A SNN is applied as a connected network of place cells which is producing a cognitive map and acts as a medium for a propagating wave that traverses the network with the successive activation of different neurons; (2) A control process supported by STDP, a temporally asymmetric learning rule induced by temporal correlations between the spikes of pre and post synaptic neurons, is introduced to change the synaptic connectivity within the network and to stabilize the place cell network; (3) A vector field converging on the target locations is generated by the updated synaptic connectivity weights, which enables the computation of a feasible path from the starting point to the target.

### 3.1. Neuron model

Each place cell is an excitatory neuron representing a part of the environment called the place cell field. The initial connections between neurons are modeled as synapses whose weights are inversely proportional to the distance between the current neuron and neighboring neurons. A probate method for modeling place cells is the Gaussian distribution, which can represent the firing of place cells but does not provide a basis for physiological message transmission. To better consider and rely on physiological phenomena, SNNs are introduced to model place cells in analogy to the physiology of cellular information transmission. SNNs are characterized by a spiking neuron model, and one widely used model is the LIF model (Rast et al., 2010; Zennir et al., 2015). Therefore, the LIF model is adapted to model the place cells. The membrane potential of place cells can be expressed as Ponulak and Hopfield (2013).


(1) $$\begin{array}{l} \tau_{m}\frac{du_{m}\left(t\right)}{dt}=-\left(u_{m}\left(t\right)-u_{r}\right)+R_{m}I_{m} \end{array}$$

Where *u*_*m*_(*t*) represents the membrane potential of the place cells; τ_*m*_ indicates the membrane time constant; *u*_*r*_is the resting membrane potential; *R*_*m*_describes the membrane resistance; *I*_*m*_stands for the total synaptic current and can be written as,


(2) $$\begin{array}{l} I_{m}=i_{sens}\left(t\right)+i_{syn}\left(t\right)+i_{ns}\left(t\right)-i_{inh}\left(t\right)-i_{Ca}\left(t\right) \end{array}$$

Where *i*_*sens*_(*t*) is the sensory input, *i*_*syn*_(*t*) is a sum of the currents delivered by the individual excitatory synapses entering the given neuron, *i*_*ns*_(*t*) is the non-specific background current modeled as a Gaussian process with mean zero and variance 5 nA, *i*_*inh*_(*t*) is the global inhibitory current, *i*_*Ca*_(*t*) represents a neuron- specific inhibitory current that could be activated by calcium-activated potassium channels in real neurons and can be calculated as,


(3) $$\begin{array}{l} \tau_{Ca}\frac{di_{Ca}\left(t\right)}{dt}=-i_{Ca}\left(t\right) \end{array}$$

**Figure 3:**
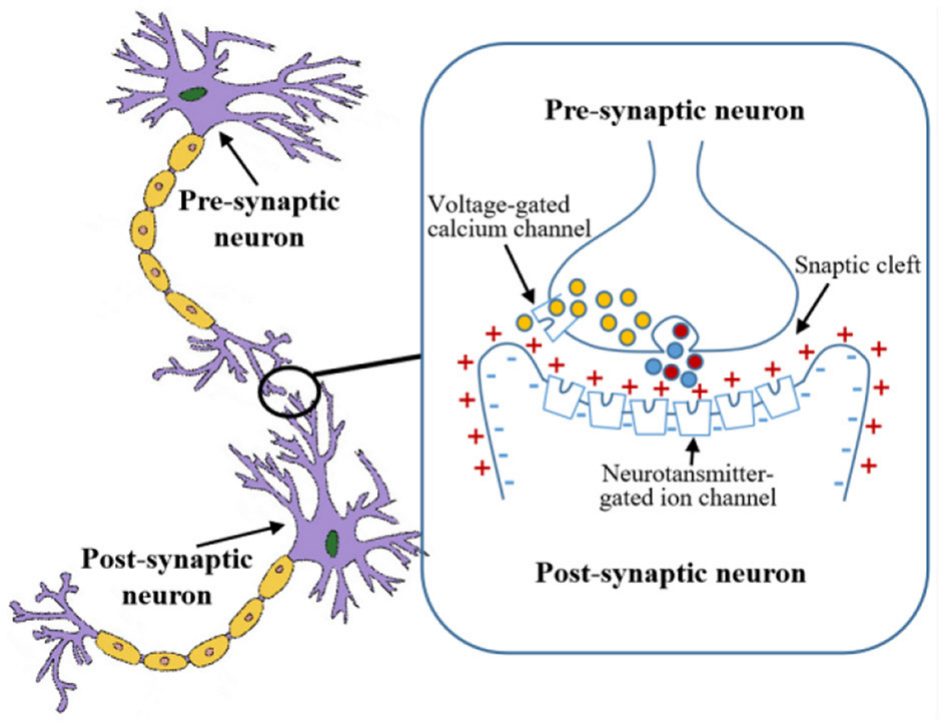
Neuron model of place cells.

The ordinary differential equation describes a spiking neuron as a dynamic system in which the membrane potential is accumulated. When the membrane potential from the input signal reaches a certain threshold, the place cell emits a pulse, called a spike. The presence of spikes enables asynchronous communication between neurons. The LIF model of place cells is consistent with the biological neuronal synaptic information transmission mechanism. Namely, the pre-synaptic neuron releases neurotransmitters which open ion channels on the membrane of the post-synaptic neuron. The neuron model is shown in Figure 3.

### 3.2. Neural plasticity-based weight update

Place cell model training plays a critical role for autonomous drone trajectory planning. STDP is a biological process related to SNNs that is thought to be responsible for learning and memorizing information in the brain (Izhikevich, 2007). This process provides a biologically plausible explanation for the activity-dependent development of nerves in terms of long-term potentiation and long-term depression, which is suitable for training the weights of the synaptic connections between place cell neurons. Suppose place cell *x*_*i*_ is connected to place cell *x*_*j*_ by a synapse running from *x*_*i*_ to, with weight *w*_*ij*_. When *x*_*i*_ fires before *x*_*j*_, STDP is assigned to increase the synaptic weight between the neurons, and when *x*_*i*_ fires after *x*_*j*_, the synaptic weight between these two neurons decreases. The synaptic connections are changed according to the STDP model described by the following equation (Ponulak and Hopfield, 2013).


(4) $$\begin{array}{l} \frac{dw_{ji}\left(t\right)}{dt}=a+d\\ \left[S_{i}\left(t\right)\overset{\infty }{\underset{0}{\int }}a_{ij}\left(s\right)S_{i}\left(t-s\right)ds+S_{j}\left(t-s\right)\overset{\infty }{\underset{0}{\int }}a_{ji}\left(s\right)S_{i}\left(t-s\right)ds\right] \end{array}$$

Where *a* < 0 denotes the activity-independent weight loss; *S*_*i*_(*t*) and *S*_*j*_(*t*) represent the presynaptic and postsynaptic spike trains, respectively. A spike train is defined as $S\left(t\right)=\sum_{f}\delta \left(t^{f}-t\right)$, where *t*^*f*^ is the f-th firing time. The terms*a*_*ij*_(*s*) and *a*_*ji*_(*s*) are the integral kernels, where *s* is the delay between the presynaptic and postsynaptic firing times $s=t_{i}^{f}-t_{j}^{f}$.

The kernels *a*_*ij*_(*s*) and *a*_*ji*_(*s*) determine the shape of the STDP learning window and are defined as follows (Mahadevuni and Li, 2017).


(5) $$\{\begin{array}{l} a_{ji}(-s)=+A_{ji}\cdot \exp(s/\tau_{ji}\;)\;if\;s\leq 0\\ a_{ij}(-s)=+A_{ij}\cdot \exp(-s/\tau_{ij}\;)\;if\;s>0 \end{array}$$

**Figure 4:**
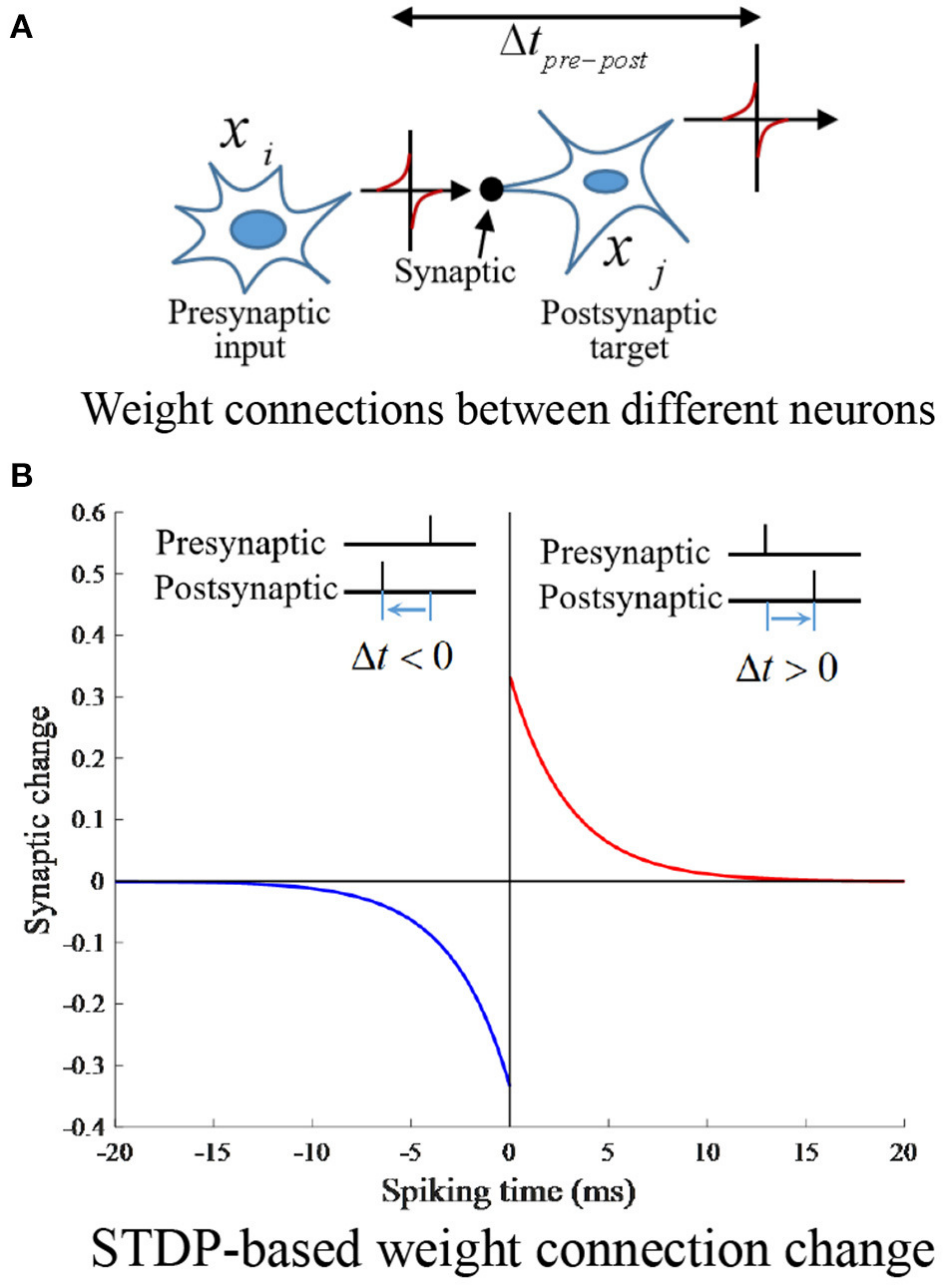
STDP connection weights between place cells. **(A)** Weight connections between different neurons. **(B)** STDP-based weight connection change.

Where *A*_*ji*_, *A*_*ij*_ depict the amplitudes; τ_*ji*_, τ_*ij*_ denote the time constants of the learning window and *A*_*ji*_>*A*_*ij*_>0, τ_*ji*_ = τ_*ij*_>0. The STDP connection weights between place cells is shown in Figure 4.

### 3.3. Optimal path finding

The SNN can be seen as an interconnected network of place cells that forms a cognitive map and is a medium for a propagating wave that traverses the network with the successive activation of different neurons. The propagating wave indirectly carries information about distance by generating a synaptic vector field. In the proposed model, obstacles and dangerous regions are considered as place cells that are inhibited by the prefrontal cortex. An inhibited neuron in the SNN cannot serve as a medium for the propagating wave. Consequently, all future paths will pass by the inhibited neurons (obstacles). For each place cell *n*_*i*_ in the network, we consider a set *N*_*ji*_ of all neurons *n*_*j*_ to which *n*_*i*_makes direct synaptic projections. For the place cell *n*_*i*_, the synaptic vector field *r*_*i*_(*t*) is defined according to the connection weights of the place cell (Steffen et al., 2020).


(6) $$\begin{array}{l} r_{i}\left(t\right)=\underset{j}{\sum }w_{ji}\left(t\right)\left(x_{j}-x_{i}\right)/\underset{j}{\sum }w_{ji}\left(t\right) \end{array}$$

Where *x*_*i*_ and *x*_*j*_ represent the current position of the drone and the center of the place cell field.

Suppose the vector *r*_*i*_(*t*) starts at the current location *x*_*i*_ of place cell *n*_*i*_ and ends at a centroid of the center of the place cell field *x*_*j*_ of the neighboring place cells *n*_*j*_∈*N*_*ji*_, weighted by the corresponding connection weights *w*_*ji*_(*t*). Then, the drone is driven by the synaptic vector field.


(7) $$\begin{array}{l} V_{speed}\left(t+1\right)=V_{speed}\left(t\right)+{\left[V_{force}\left(t\right)+r_{i}\left(t\right)\right]}^{*}S \end{array}$$

**Figure 5:**
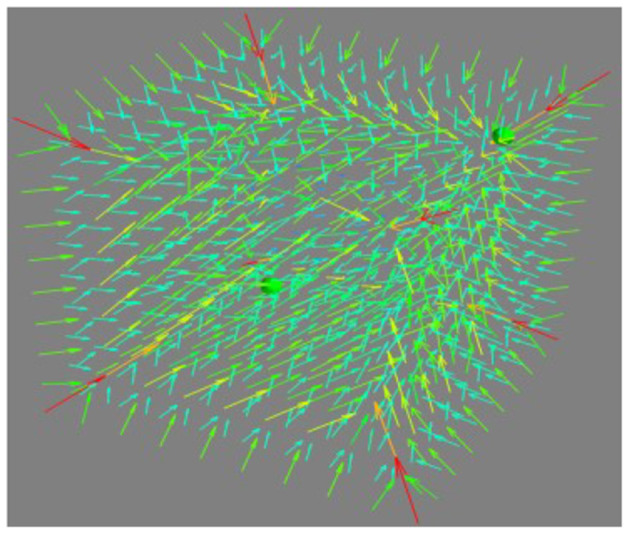
The synaptic vector field.

Where *S* is the smoothing factor in the range between zero and one. The synaptic vector field is shown in Figure 5.

At the same time, obstacles and dangerous locations are considered by place cells inhibited by the prefrontal cortex. An inhibited neuron in the SNN cannot transport the propagating wave. Consequently, all future paths will avoid the inhibited neurons. Finally, the current position of the drone can be updated as,


(8) $$\begin{array}{l} P\left(t+1\right)=P\left(t\right)+V_{speed}\left(t+1\right) \end{array}$$

Where *P*(*t*) and *P*(*t*+1) are the position of the drone at time *t* and time *t*+1, respectively; *V*_*speed*_ is the velocity vector. After training, the drone is able to find a feasible path to the target. The pseudocode for the brain-inspired path planning algorithm is shown in Algorithm 1.

**Algorithm 1:** Generating a feasible path.

1: **Input:** Start position *P*_0_; Target position *P*_*T*_;
2: **Output:** *Path*←*a*set of waypoint
3: **for** *t* ≤ *T*_max_ **do**
4: compute *u*_*m*_ by Eq. (1);
5: compute *w*_*ji*_(*t*) by Eq. (4);
6: compute *r*_*i*_(*t*) by Eq. (6);
7. compute *V*_*speed*_(*t*+1) by Eq. (7);
8: update *P*(*t*+1) by Eq. (8);
9: **if** find the target
10: **exit**
11: **end if**
12: **end for**
13: **return** *P*(*t*+1)←*P*(*t*)+*V*_*speed*_(*t*)

## 4. Results

To investigate the effectiveness and the feasibility of the path planning method for drone operations, experiments have been conducted on simulation basis with a given environment supported by Airsim, which is based on a model of the flying engine.

### 4.1. Simulation experiments

In the first part of the experiments, the brain-inspired path planning algorithm is evaluated in simulation-only environments. The simulations were performed on a computer with a 1.60 GHz Core I5 processor and 16 GB RAM and implemented using the Python language and the Nengo package. The ranges of the environment along the axes OX, OY, and OZ are [0, 30], [0, 30], and [0, 30] m respectively. The start position and the target position are set to (0, 0, 0) and (26, 26, 26), respectively. Our goal is to enable the drone to find a short and collision-free path from the starting point to the target. The spiking neural network was trained using the STDP learning rule. The parameters for spiking neural network and STDP weight training were all determined empirically, as in Table 1. The parameters chosen are the same for all experiments.

**Table 1:** Parameter setting.

**Parameter**		**Value**
Membrane time constant	τ_*m*_	0.002
Rest membrane potential	*u*_*r*_	0 mV
Membrane resistance	*R*_*m*_	20 M
Pre-amplitude	*A*_*ji*_	1.0
Post-amplitude	*A*_*ij*_	1.0
Pre-time constant	τ_*ji*_	0.0168
Post-time constant	τ_*ij*_	0.0337

**Figure 6:**
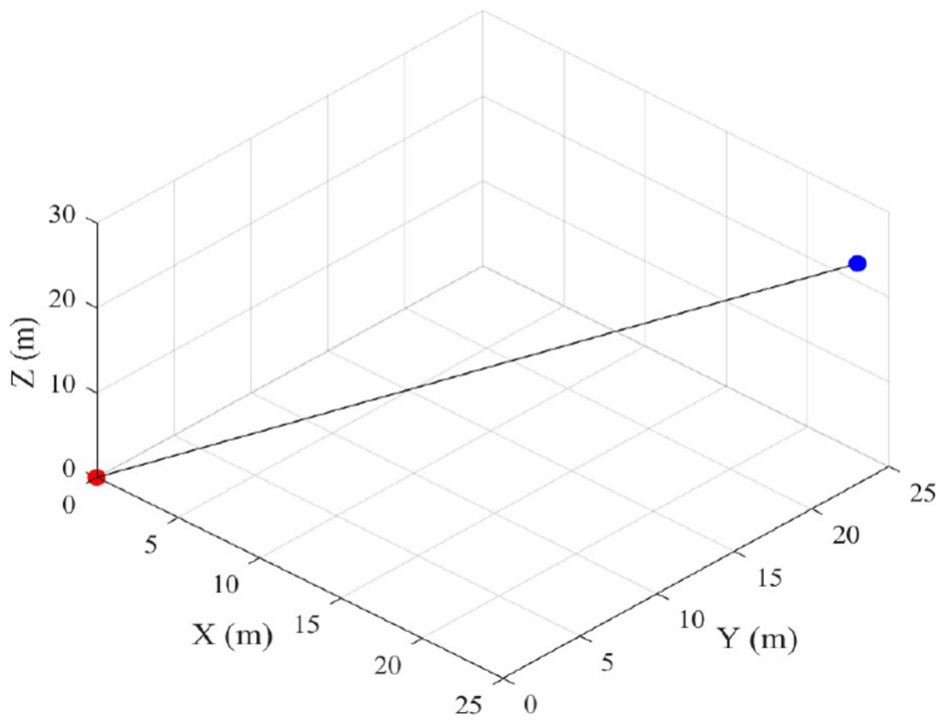
3D path without obstacle.

After training, a vector field is generated to guide the drone to the target. Finally, based on the proposed method, the most cost-effective path can be generated. Two types of environments were tested. First, a simple 3D environment without obstacles was considered. The simulation result is shown in Figure 6, in which a path is shown between the red starting point and the blue target position. The figure shows, that the drone is able to find a direct path between both points, using the proposed algorithm.

**Figure 7:**
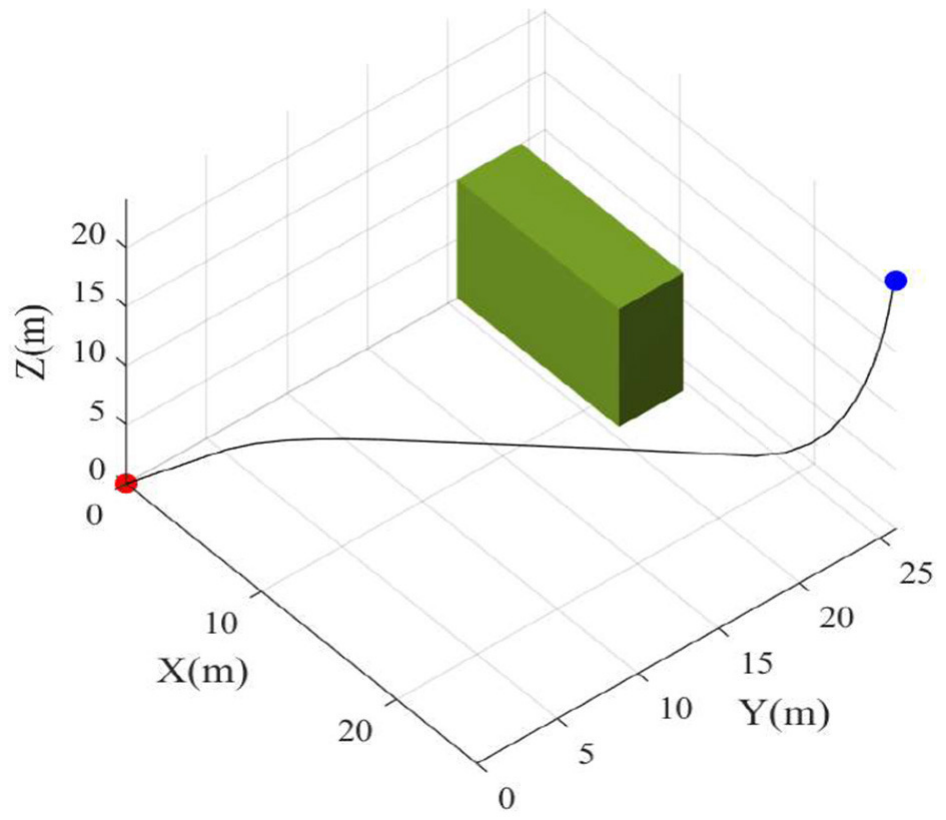
3D path with obstacle.

Second, an environment with an obstacle is utilized to verify the performance of the algorithm. As shown in Figure 7, the drone can avoid obstacles in its path and find a collision-free path.

Both experiments in the Python simulation environment show that the proposed algorithm has good effect for intelligent path finding of drones. The execution time depends only on the length of the feasible path between the starting point and the target, not on the complexity of the environment.

### 4.2. Simulation in Airsim

To further verify the pathfinding capability of the proposed algorithm, the high-fidelity drone simulator Microsoft Airsim (https://microsoft.github.io/AirSim/; Shah et al., 2018) has been applied for this purpose. Microsoft Airsim acts as a plugin for the Unreal Engine, a 3D computer graphics game engine. The Unreal Engine can render photorealistic environments and provide features such as different flight conditions, realistic scenes, physical collision effects, etc. These features make the combination of Airsim and Unreal Engine a promising toolset for drones performing various flight tasks. Meanwhile, Visual Studio is applied to compile and launch Unreal Engine project files.

**Figure 8:**
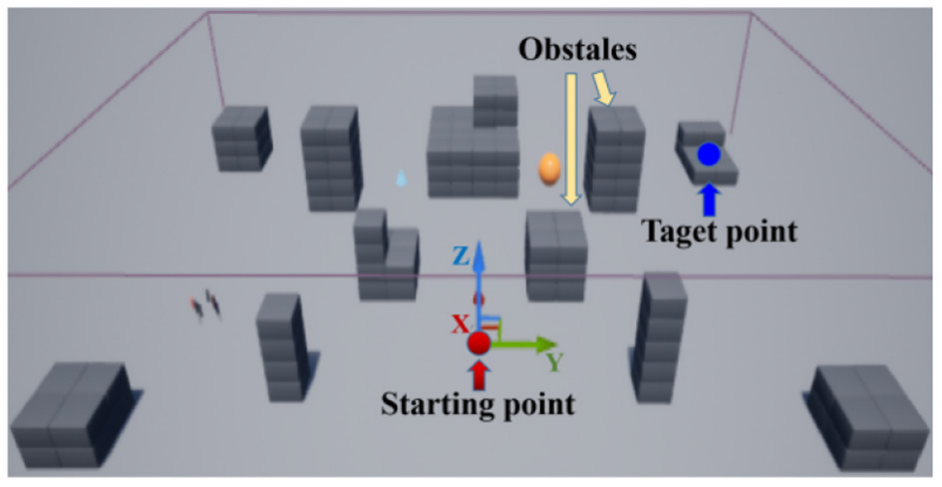
Flight environment settings in Airsim.

In Unreal Engine, there are many pre-built scenes in the application store that include blocks, landscapes, forests and so on. Airsim also provides users with rich interfaces that facilitate the control of the drone. In this research, the default environment “blocks” is utilized. The area of the map is 150^*^230^*^25 m. Since the Unreal Engine keeps track of all the objects displayed in the scene, it can easily retrieve the state of the drone and the pose of each object through APIs. The setting of the environment is shown in Figure 8. In the environment, the positions of the red dot and the blue dot are set as the starting point and the target point, respectively. The gray blocks of the considered as obstacles.

**Figure 9:**
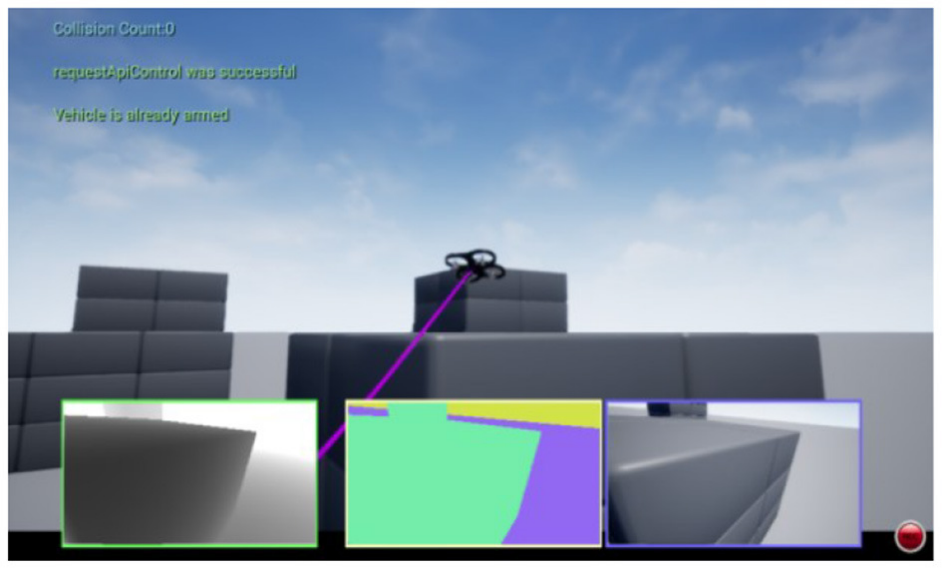
Real flight scene.

After setting the environment, the drone is trained using the method proposed in Section 3. Then a path can be generated. Figure 9 shows the realistic scenario of the flying drone with the help the Unreal Engine animation program. The three sub-windows in the lower part of Figure 9 present the depth image, segmentation view (Zhao et al., 2022), and RGB view (Xia et al., 2022) of the on-board camera during flight.

**Figure 10:**
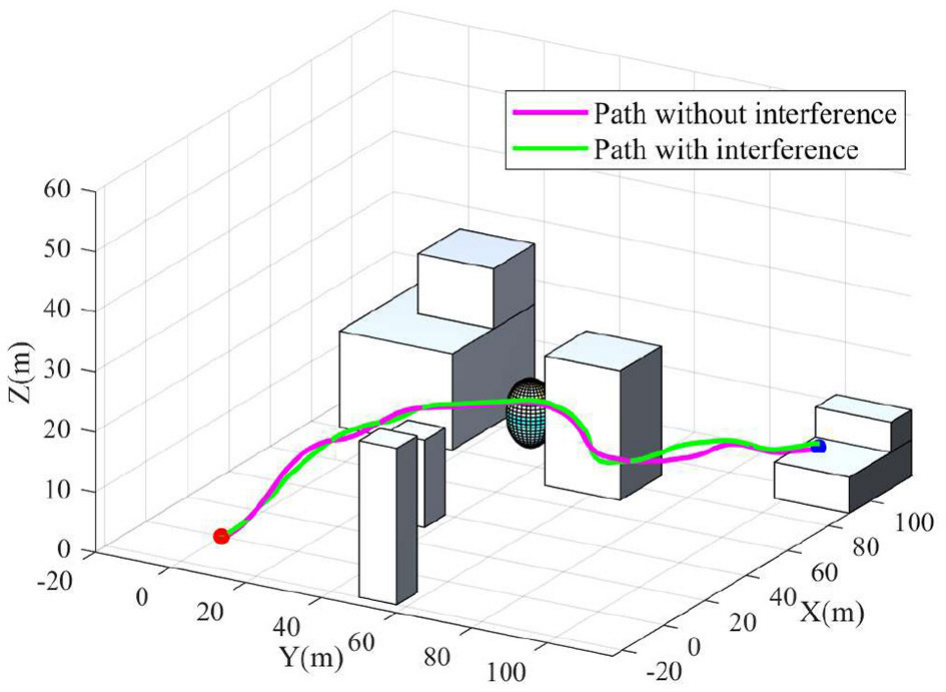
Scale map in Airsim and path comparison.

To get a better overview of the drone flight in Airsim, part of the environment and the flight path are extracted in a scaled sketch, as shown in Figure 10. In the figure, the red dot represents the starting position; the blue dot denotes the destination point; the purple line is the path generated by the proposed algorithm. The drone is capable to find a short path from the starting point to the target point without any collisions, which confirms the capability of the proposed algorithm. The STDP training process of the spiking neural network takes 2.59 min, and the whole path finding process takes 3.49 min. The simulation experiment with Airsim also shows that the algorithm has a great advantage in terms of path finding speed. In addition, the computationbal speed will be significantly improved if a GPU or neuromorphic hardware is to be applied. In order to further verify the robustness of the algorithme, wind speed interference is added in Airsim simulator. The green path in Figure 10 is the path generated when this interference is added. It can be seen that the drone can still find the target successfully after adding interference, and the path does not fluctuate sicnificantly, which underlines the robustness of the proposed method.

**Figure 11:**
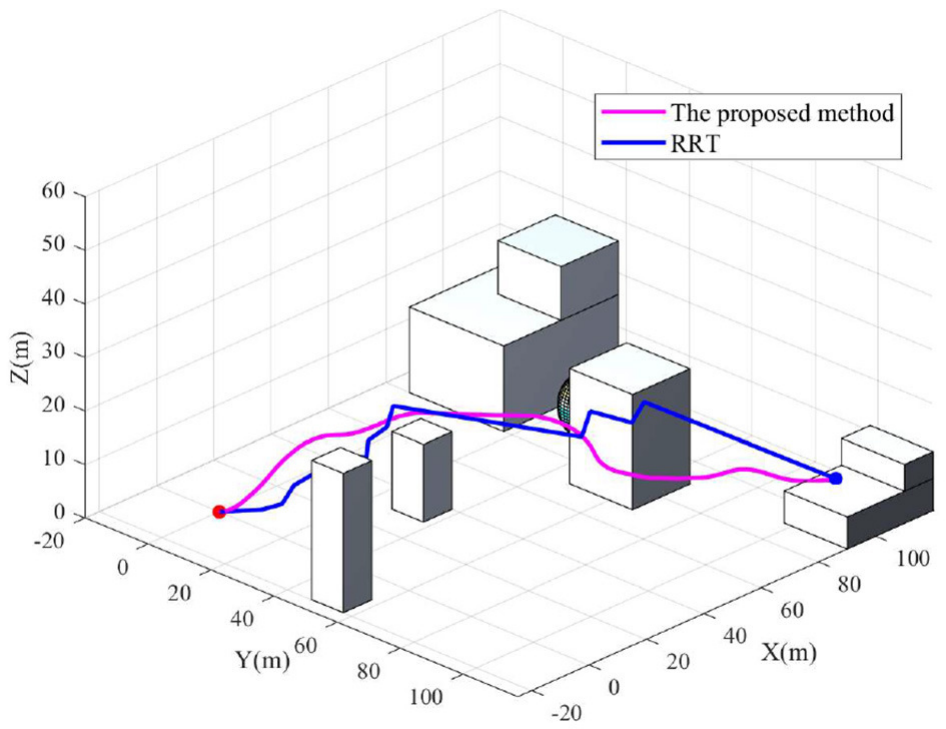
Path comparison between the proposed algorithm and RRT algorithm.

To further demonstrate the performance of the proposed method, comprehensive comparison with related algorithms is carried out. Among the most prominent path planning methods, RRT is one of the most promising and widely used methods. Therefore, the proposed method in this paper is compared with the RRT algorithm. The path comparison result is shown in Figure 11.

It can be seen from Figure 11 that the path generated by RRT does not conform to the optimal flight path of a drone. However, the path obtained by the proposed algorithm in this paper can generate a smooth path which is more suitable for the flight of a drone while ensuring the arrival of the target.

## 5. Discussion

A new approach for planning the trajectory of unmanned multicopter drones with obstacles based on SNN is described. The proposed method relies on the concept of propagating waves in a cognitive map to find a feasible and collision-free path. The computational complexity depends on the distance and resolution of the map. The unique feature of this method is that the considered neuron shows close analogy to the biological neurons. In our proposal, the wave is stabilized by exploiting the natural plasticity process of biological synapses, which regulates the synapse weight depending on the spiking time of the presynaptic and the postsynaptic neuron. The experimental results show that the proposed planning method can be used for routing a robust path of a drone flight in different environmental conditions.

Compared to related approaches, the described approach has the following proposing characteristics:

1. A biologically plausible path planner that equips the drone with animal-like behavior, improving the decision-making intelligence of the drone.
2. LIF is implemented to model the membrane potential of place cells to represent the spatial environment, which corresponds to the physiological mechanism of information transfer between neurons.
3. A neuroplasticity based learning process, STDP, is applied to update the weights between neurons and select the optimal action that enables the synaptic vector field to steadily guide the drone to its target.

It is worth noting that the path generated by the proposed method is not the geometrically shortest path, but a smooth flight path that allows for the actual flight of the UAV while taking into account the shortest path.

In addition to relying on a physiological plausible basis, the proposed method has better robustness and allows higher computational speed compared with heuristic algorithms. And the designed algorithm in static and known environments does not need to equip a drone with model based sensors but with event cameras, which can greatly reduce the cost and speed up the path finding process.

However, the proposed algorithm also has some limitations. The drone starts flying only after the global path is generated. Therefore, the results are not real time but still robust, considering properties of the algorithm and the used processor. In addition, the main focus in this paper is to find collision-free paths for drones in static and known environment, without considering the perception and path planning in dynamic environment in some cases. Moreover, when the range of flight environment becomes larger, the computational cost increases exponentially and the path generation time becomes longer while keeping the resolution constant.

According to the above limitations, in future research, it is necessary to consider equipping Airsim with dynamic sensing sensors such as event cameras to capture the dynamic obstacles encountered in the flight environment and achieve dynamic obstacle avoidance in unknown environments. Moreover, the proposed algorithm needs to be combined with a dedicated brain-like chip to improve the computing performance through high- performance hardware and to be applied in practice.

## Data availability statement

The original contributions presented in the study are included in the article/Supplementary material, further inquiries can be directed to the corresponding author.

## Author contributions

All authors listed have made a substantial, direct, and intellectual contribution to the work and approved it for publication.
